# Supplementary material for: A closely related pair of superoxide dismutase isozymes from Staphylococcus aureus show distinct stabilities and proton-exchange dynamics
Source: J Biol Chem. 2026 Jun 5;302(7):113226. doi: 10.1016/j.jbc.2026.113226 (PMC13321213; doi:10.1016/j.jbc.2026.113226)
Supplement: Supporting-information [file mmc2.pdf]

SUPPORTING INFORMATION

A closely related pair of superoxide dismutase isozymes from *Staphylococcus aureus* show distinct stabilities and proton-exchange dynamics

Mariam Esmaeeli[1†], Lorna Nikolić[1,2†], Rafał Mazgaj[1], Swati Das[1,2], Lilia Zhukova[1], Weronika Puchała[1], Michał Dadlez[1], and Kevin J. Waldron[1]*

[1] Institute of Biochemistry & Biophysics, Polish Academy of Science, Warsaw 02-106, Poland

[2] Doctoral School of Molecular Biology and Biological Chemistry, Institute of Biochemistry and Biophysics, Polish Academy of Science, Warsaw 02-106, Poland

[†] These authors contributed equally

* Correspondence:

kwaldron@ibb.waw.pl: Tel: + 48 225 923 342

Working title: Structural stability differences between Staphylococcal SODs

Keywords: Superoxide dismutase; protein stability; protein evolution; hydrogen-deuterium exchange; circular dichroism

Supplementary Figures:

```
EcSodA_FM1_M      -----------------------MSYTLPSLPYAYDALEPHFDKQTMEIHHTKHHQTYVN   37
SacamSOD_FM1_C    -----------------------MAFKLPNLPYAYDALEPYIDQRTMEFHHDKHHNTYVT   37
SaMnSOD_FM1_M     -----------------------MAFELPKLPYAFDALEPHFDKETMEIHHDRHHNTYVT   37
Cfas_FM2_M        ----------------------MREIVLPPLPYAENELEPYISAQTVMLHYGKHLKGYID   38
EcSodB_FM2_F      -----------------------MSFELPALPYAKDALAPHISAETIEYHYGKHHQTYVT   37
Bfra_FM2_C        ------------------------MYEMPKLPYANNALEPVISQQTIDYHYGKHLQTYVN   36
Mabs_FM3_M        ----------------------MAEYTLPDLDYDYGALEPHISGQINELHHSKHHATYVK   38
Mtub_FM3_F        ----------------------MAEYTLPDLDWDYGALEPHISGQINELHHSKHHATYVK   38
Nomu_FM4_M        ----------------MKKFE-EMKFNIPELK-------G-ISKKNID-EHLKLYAGYVK   34
Afer_FM4_F        ----------------MSEYSVREELKPSGLD-------G-ISDAQIN-DHWGLYAGYVN   35
Paca_FM5_M        ---------------------MTLQYKLPDLPYDLGDLEPVISKEIMSLHYNKHHATYVA   39
Hsap_FM5_M        MLSRAVCGTSRQLAPALGYLGSRQKHSLPDLPYDYGALEPHINAQIMQLHHSKHHAAYVN   60
                                                *          :.      .:      *:

EcSodA_FM1_M      NANAALESLPEFANLPVEELITKLDQLPADKKTVLRNNAGGHANHSLFWKGLKKG-----   92
SacamSOD_FM1_C    KLNATVEGT-ELEHQSLADMIANLDKVPEAMRMSVRNNGGGHFNHSLFWEILSPN-----   91
SaMnSOD_FM1_M     KLNAAVEGT-DLESKSIEEIVANLDSVPANIQTAVRNNGGGHLNHSLFWELLSPN-----   91
Cfas_FM2_M        NVNRLIKEISNLEGKDLETIVKQ-------SEGSLYNNAAQAWNHIFYFDAFSPH---AQ   88
EcSodB_FM2_F      NLNNLIKGT-AFEGKSLEEIIRS-------SEGGVFNNAAQVWNHTFYWNCLAPN---AG   86
Bfra_FM2_C        NLNSLVPGT-EYEGKTVEAIVASA------PDGAIFNNAGQVLNHTLYFLQFAPKP--AK   87
Mabs_FM3_M        GVNDAVAKLEEAREKGDHAAIFL-------NEKNLAFHLGGHVNHSIWWKNLSPN---GG   88
Mtub_FM3_F        GANDAVAKLEEARAKEDHSAILL-------NEKNLAFNLAGHVNHTIWWKNLSPN---GG   88
Nomu_FM4_M        NANLITDKIGEYMADPEKNMFVL-----GELQRRFSFEFNGIRNHEYYFASLEGGAKP--   87
Afer_FM4_F        QSNALRKELAEMRAAGKTGSLTY-----ADRRRRFGFEYNGMVLHEYYFAQLKPGSSI--   88
Paca_FM5_M        NLNKALEQYAEAEAKNDLPALIT-------LQSAINFNGGGHINHSIFWTNLAPQKSGGG   92
Hsap_FM5_M        NLNVTEEKYQEALAKGDVTAQTA-------LQPALKFNGGGHINHSIFWTNLSPN---GG   110
                    *                               .  .      *  ::  :

EcSodA_FM1_M      TTLQGDLKAAIERDFGSVDNFKAEFEKAAASRFGSGWAWLVLK-G-DKLAVVSTANQDSP   150
SacamSOD_FM1_C    SEEKGGVIDDIKAQWGTLDEFKNEFANKATTLFGSGWTWLVVN-D-GKLEIVTTPNQDNP   149
SaMnSOD_FM1_M     SEEKGTVVEKIKEQWGSLEEFKKEFADKAAARFGSGWAWLVVN-N-GQLEIVTTPNQDNP   149
Cfas_FM2_M        HLPSGNLEKQINKQWGNFENFKTAFTNTANNLFGSGWVWLIKNKN-GTLDIIGESNAGNV   147
EcSodB_FM2_F      GEPTGKVAEAIAASFGSFADFKAQFTDAAIKNFGSGWTWLVKNSD-GKLAIVSTSNAGTP   145
Bfra_FM2_C        NEPAGKLGEAIKRDFGSFENFKKEFNAASVGLFGSGWAWLSVDKD-GKLHITKEPNGSNP   146
Mabs_FM3_M        DKPTGDLAAAIDDQFGSFDKFQAQFTAAANGLQGSGWAVLGYDSLGQKLLTFQLYDQQA-   147
Mtub_FM3_F        DKPTGELAAAIADAFGSFDKFRAQFHAAATTVQGSGWAALGWDTLGNKLLIFQVYDHQT-   147
Nomu_FM4_M        LSANSKLKTAIENQTPSFDAWLVSFKLLAMT-RGVGWAVLAWDHDTEQFLHIWVDEQHLG   146
Afer_FM4_F        -DQAPGFKAAVTEQFGSTEAWHEDLMSAAKS-RSVGWAISYYDGTTGQINNHFVQLHEDG   146
Paca_FM5_M        APPEGAIAEAIQKQFGSLEKFIETMNTKTAAIQGSGWGWLGYCKNKKQLEIVTCSNQDPL   152
Hsap_FM5_M        GEPKGELLEAIKRDFGSFDKFKEKLTAASVGVQGSGWGWLGFNKERGHLQIAACPNQDPL   170
                        .   :     .   :   :   :    . **           :

EcSodA_FM1_M      LMGEAISGASGFPIMGLDVWEHAYYLKFQN-RRPDYIKEFWNVVNWDEAAARFAAKK---   206
SacamSOD_FM1_C    LTE------GKTPILLFDVWEHAYYLKYQN-KRPDYMTAFWNIVNWKKVDELYQAAK---   199
SaMnSOD_FM1_M     LTE------GKTPILGLDVWEHAYYLKYQN-KRPDYIGAFWNVVNWEKVDELYNATK---   199
Cfas_FM2_M        LKG------NGKPLLGVDIWEHAYYLDYQN-RRAEHIDGLWNIIDWSIVDRRYNE-----   195
EcSodB_FM2_F      LTT------DATPLLTVDVWEHAYYIDYRN-ARPGYLEHFWALVNWEFVAKNLAA-----   193
Bfra_FM2_C        VRA------GLKPLLGFDVWEHAYYLDYQN-RRADHVNKLWEIIDWDVVEKRL-------   192
Mabs_FM3_M        NVP-----LGIIPLLQVDMWEHAFYLQYKN-VKADYVKAFWNVVNWADVQDRYTAATTKT   201
Mtub_FM3_F        NFP-----LGIVPLLLLDMWEHAFYLQYKN-VKVDFAKAFWNVVNWADVQSRYAAATS--   199
Nomu_FM4_M        QLN------GACWILAIDMWEHAFVYDYPTSEKKKYIEAFFENLNWEVAEEYFTELQ---   197
Afer_FM4_F        NVG------GFVPLVIVDVFEHAYMVDWKALGRADYLAAIHKNMNWGVVEARFQAAKSGQ   200
Paca_FM5_M        A-A-----KGLVPLLGIDVWEHAYYLQYKN-VRADYLKAIWNIVNWKNVEERFTKAKAE-   204
Hsap_FM5_M        QGT-----TGLIPLLGIDVWEHAYYLQYKN-VRPDYLKAIWNVINWENVTERYMACKK--   222
                           .   :: .*::***:  .:    :  .   :   ::*  .
```

Supplementary Figure S1: Annotated alignment of SodFM sequences. Annotated partial sequence alignment of selected SodFM isozymes, from all five sub-families, illustrating important conserved residues. The pair of isozymes under study herein from *Staphylococcus aureus* (*Sa*MnSOD and *Sa*camSOD), highlighted with a grey background, which both belong to subfamily SodFM1, were aligned with the SodFM1 enzyme from *Escherichia coli* (*Ec*SodA), three members of the SodFM2 subfamily with divergent metal-preferences, from *E. coli* (*Ec*SodB), *Coprobacter fastidiosus* (*Cfas*) and *Bacteroides fragilis* (*Bfra*), two members of the SodFM3 subfamily with divergent metal-preferences, from *Mycobacterium abscessus* (*Mabs*) and *Mycobacterium tuberculosis* (*Mtub*), two members of the SodFM4 subfamily with divergent metal-preferences, from *Nomubacteria* (*Nomu*) and *Acidithiobacillus ferrooxidans* (*Afer*), and from two members of the SodFM5 subfamily from divergent biological sources, from *Parachlamydia acanthamoeba* (*Paca*) and from *Homo sapiens* (*Hsap*). The alignment was performed with Clustal Omega. The metal-preference of each isozyme is indicated by a letter (M = manganese-preferring; F = Fe-preferring; C = cambialistic) [1]. Residues highlighted in green are the conserved metal-coordinating ligands; those in red text are the seven Trp residues found in (and conserved between) the pair of SodFMs from *S. aureus*, with the most highly conserved highlighted in yellow; in blue, the residues located at the dimer interface, which are indicated in Fig. 5 and in the main text of the manuscript; and highlighted in red are the X_{D-2}/X_{D-1} residues in the secondary coordination sphere, previously shown to influence metal-preference [5].

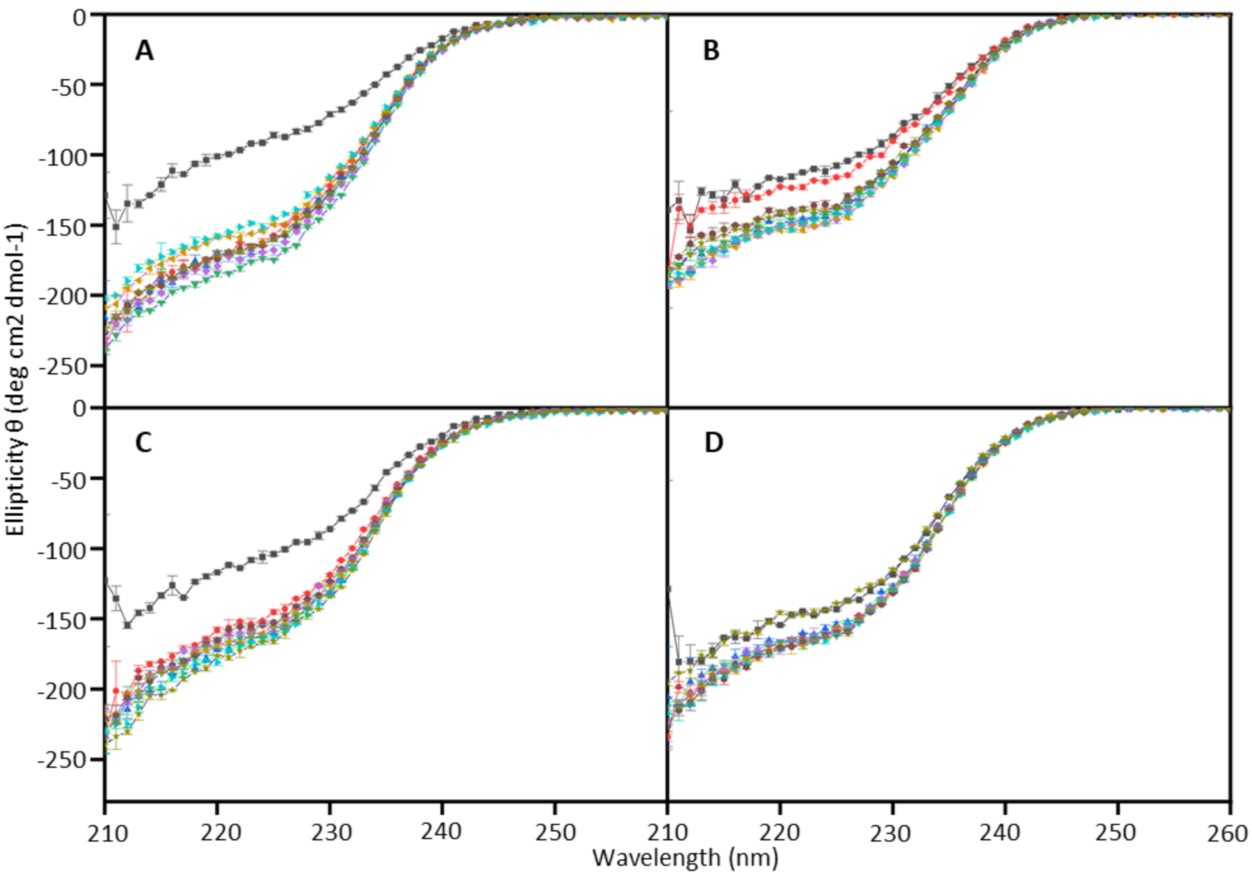

Supplementary Figure S2: CD spectroscopy shows differential unfolding of *S. aureus* SODs in urea. Complete CD spectra, data from which were used to produce the graphs shown in Fig. 2 and in Supp. Fig. S4. CD signals of 10 µM protein samples in 50 mM potassium phosphate buffer, pH 7.5, were recorded on a Jasco J-815 circular dichroism spectropolarimeter using 1 mm quartz cuvettes. Samples of each protein (A. Mn-loaded MnSOD was determined to contain 0.983 mole equivalent Mn, 0.020 Fe, 0.001 Zn; B. Fe-loaded MnSOD was determined to contain 0.990 mole equivalent Fe, 0.007 Mn, 0.000 Zn; C. Mn-loaded camSOD contained 1.070 mole equivalent Mn, 0.050 Fe, 0.00 Zn; D. Fe-loaded camSOD contained 1.090 mole equivalent Fe, 0.005 Mn, 0.000 Zn) were incubated overnight at 4 °C with different concentrations of urea (0.1 to 8 M) and then their CD spectra were over the 205 to 260 nm range

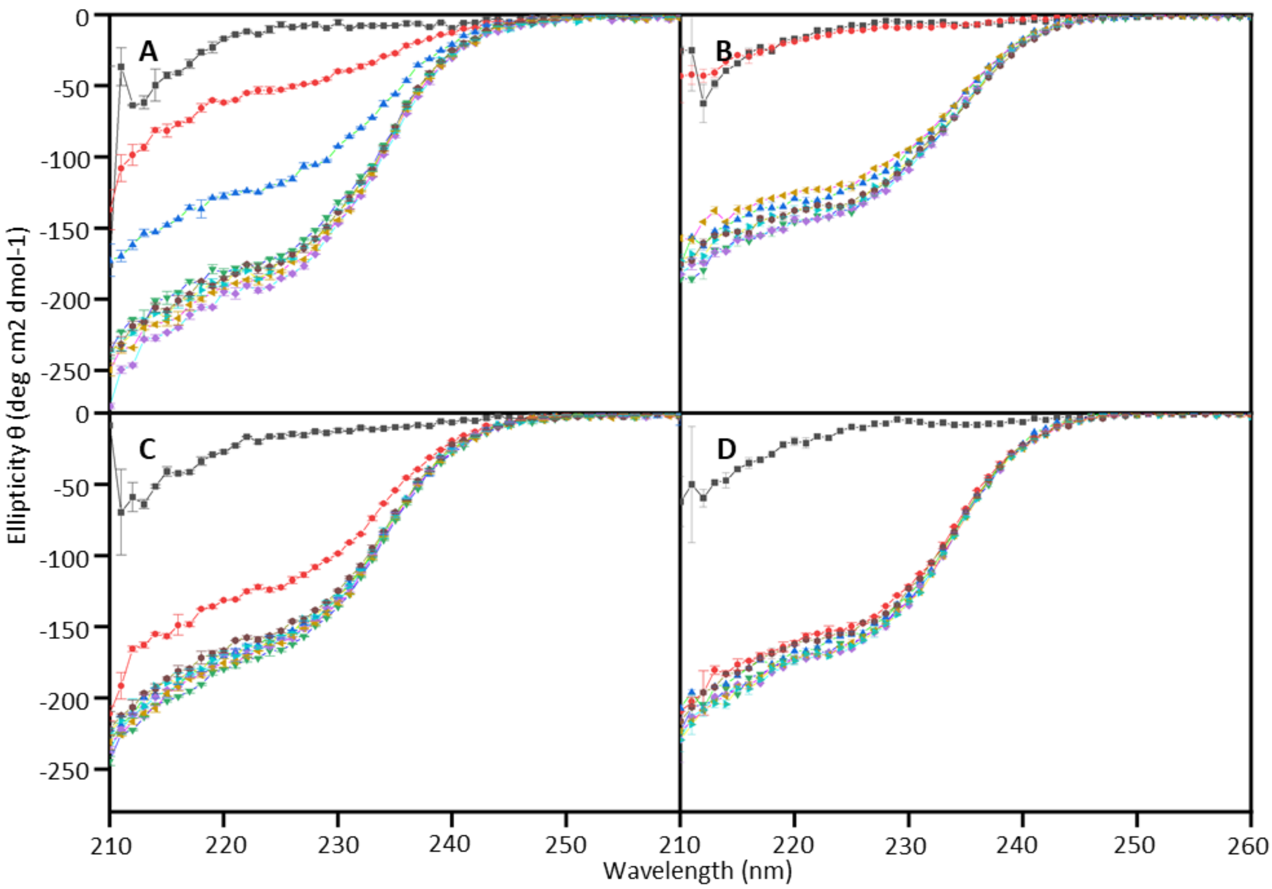

Supplementary Figure S3: CD spectroscopy shows differential unfolding of *S. aureus* SODs in guanidine. Complete CD spectra, data from which were used to produce the graphs shown in Fig. 3 and in Supp. Fig. S5. CD signals of 10 µM protein samples in 50 mM potassium phosphate buffer, pH 7.5, were recorded on a Jasco J-815 circular dichroism spectropolarimeter using 1 mm quartz cuvettes. Samples of each protein (A. Mn-loaded MnSOD; B. Fe-loaded MnSOD; C. Mn-loaded camSOD; D. Fe-loaded camSOD; metal content as described in Supp. Fig. S2) were incubated overnight at 4 °C with different concentrations of guanidine hydrochloride (0.1 to 6 M) and then their CD spectra were over the 205 to 260 nm range.

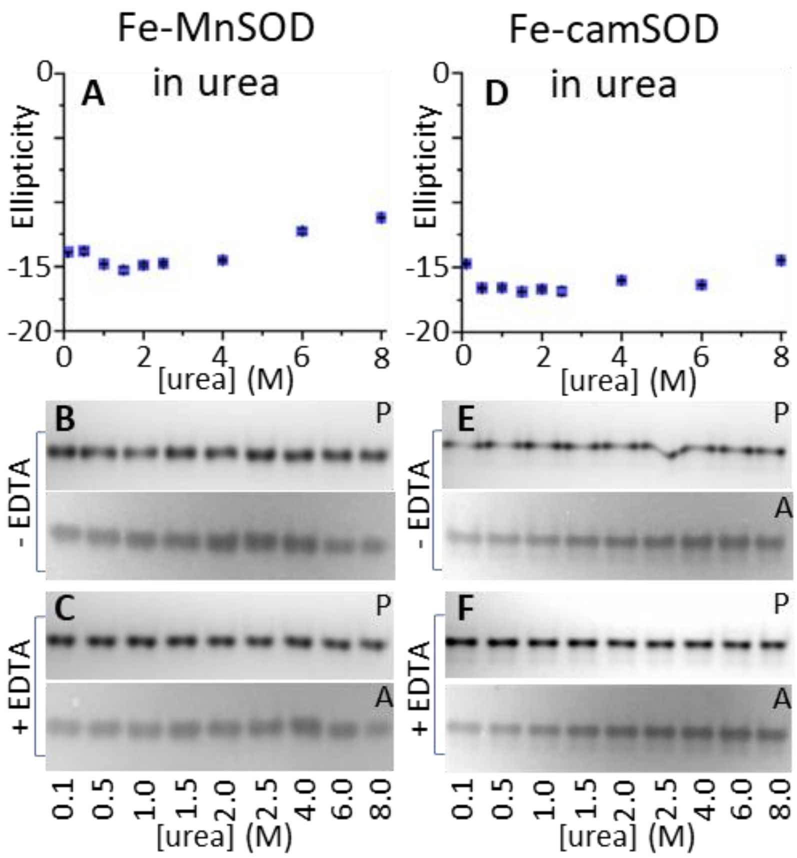

Supplementary Figure S4: CD spectroscopy shows differential unfolding of *S. aureus* SODs in urea when loaded with iron. Unfolding of the iron-loaded isoforms of (A-C) MnSOD or (D-F) camSOD by the chaotropic agent urea was assessed by (A,D) measuring their CD spectra and by assessing (B,E) protein (P) stability through Coomassie staining and (C,F) enzymatic activity (A) through NBT/riboflavin staining of native PAGE gels, either before or after incubation of the samples with EDTA. CD signals of 10 µM protein samples in 50 mM potassium phosphate buffer, pH 7.5, were recorded on a Jasco J-815 circular dichroism spectropolarimeter using 1 mm quartz cuvettes Samples of each protein (metal content as in Supp. Fig. S2) were incubated overnight at 4 °C with different concentrations of urea (0.1 to 8 M) and then their CD spectra were over the 205 to 260 nm range (Supp. Fig. S2). Each sample was measured in technical triplicates and results were presented as the average molar ellipticity (deg.cm^2.dmol^{-1}) ± standard deviation for each data point. 50 mM EDTA (5 µL) was added to aliquots (2.5 µL) of each urea-incubated sample, incubated for 2 h, then aliquots of both control and EDTA-treated urea incubated protein samples were resolved on 10 or 12% acrylamide native PAGE, and gels stained with either Coomassie Brilliant Blue for detecting protein or with NBT/riboflavin stain to detect SOD activity. For Coomassie staining, aliquots containing 280 ng protein were loaded of all samples, whereas for activity staining, aliquots containing 698.3 ng of Fe-loaded MnSOD or 41.4 ng Fe-loaded camSOD were loaded. The uncropped gel images used in this composite figure are shown in Supp. Fig. S9.

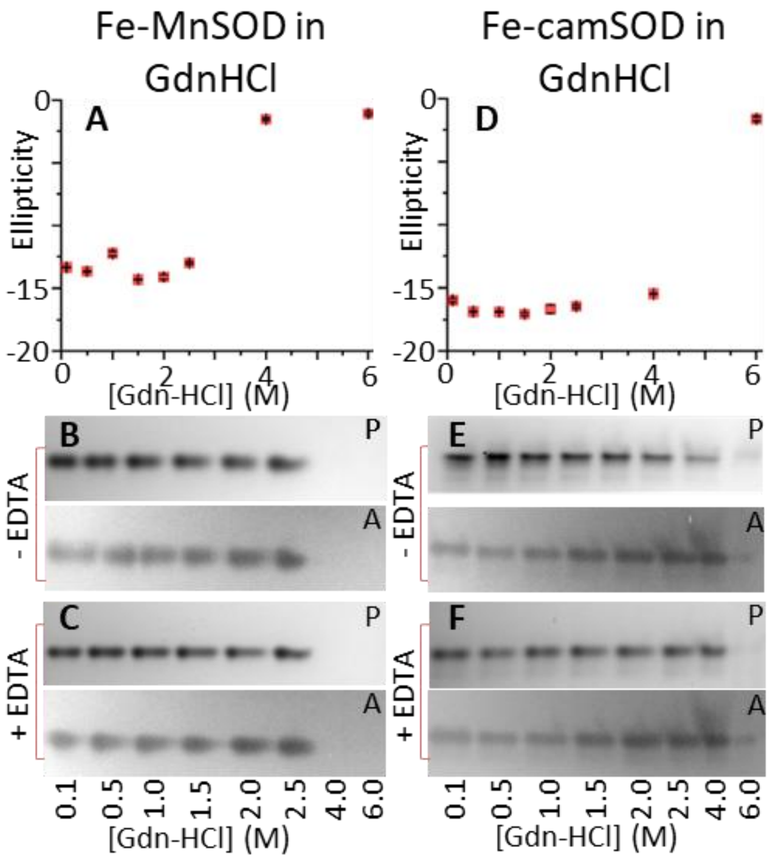

Supplementary Figure S5: CD spectroscopy shows differential unfolding of *S. aureus* SODs in guanidine when loaded with iron. Unfolding of the iron-loaded isoforms of (A-C) MnSOD or (D-F) camSOD by the chaotropic agent guanidine was assessed by (A,D) measuring their CD spectra and by assessing (B,E) protein (P) stability through Coomassie staining and (C,F) enzymatic activity (A) through NBT/riboflavin staining of native PAGE gels, either before or after incubation of the samples with EDTA. CD signals of 10 µM protein samples in 50 mM potassium phosphate buffer, pH 7.5, were recorded on a Jasco J-815 circular dichroism spectropolarimeter using 1 mm quartz cuvettes. Samples of each protein (metal content as in Supp. Fig. S2) were incubated overnight at 4 °C with different concentrations of guanidine (0.1 to 6 M) and then their CD spectra were over the 205 to 260 nm range (Supp. Fig. S3). Each sample was measured in technical triplicates and results were presented as the average molar ellipticity (deg.cm^2.dmol^{-1}) ± standard deviation for each data point. 50 mM EDTA (5 µL) was added to aliquots (2.5 µL) of each guanidine-incubated sample, incubated for 2 h, then aliquots of both control and EDTA-treated guanidine-incubated protein samples were resolved on 10 or 12% acrylamide native PAGE, and gels stained with either Coomassie Brilliant Blue for detecting protein or with NBT/riboflavin stain to detect SOD activity. For Coomassie staining, aliquots containing 280 ng protein were loaded of all samples, whereas for activity staining, aliquots containing 698.3 ng of Fe-loaded MnSOD or 41.4 ng Fe-loaded camSOD were loaded. The uncropped gel images used in this composite figure are shown in Supp. Fig. S10.

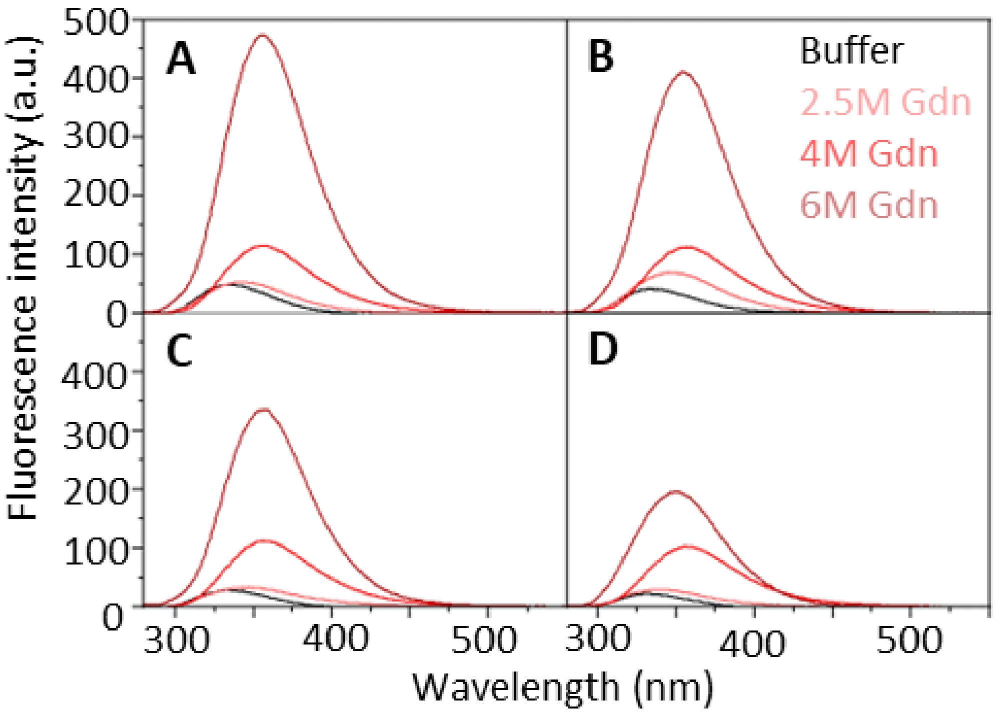

Supplementary Figure S6: Fluorescence emission of Trp residues in *S. aureus* SODs to elaborate their differential unfolding in guanidine. Trp fluorescence emission was assessed from samples (10 µM) of recombinant (A,B) MnSOD or (C,D) camSOD from *S. aureus* , loaded either with (A,C) manganese or (B,D) iron. The metal loading of the samples was as described in Fig. 1. Each sample was incubated for 24 h in either buffer (20 mM Tris, 5 mM EDTA, pH 7.5, 150 mM NaCl - black), in 2.5 M (light pink), in 4 M (intermediate pink) or in 6 M guanidine (dark pink) prior to spectra being acquired. Spectra were acquired at 550 V in 10 mm path length quartz cuvettes, spectra obtained from the respective blank solutions subtracted, and the triplicate data averaged.

Raw data uncropped gels –
Mn-loaded MnSOD unfolding in urea

Raw data uncropped gels –
Mn-loaded camSOD unfolding in urea

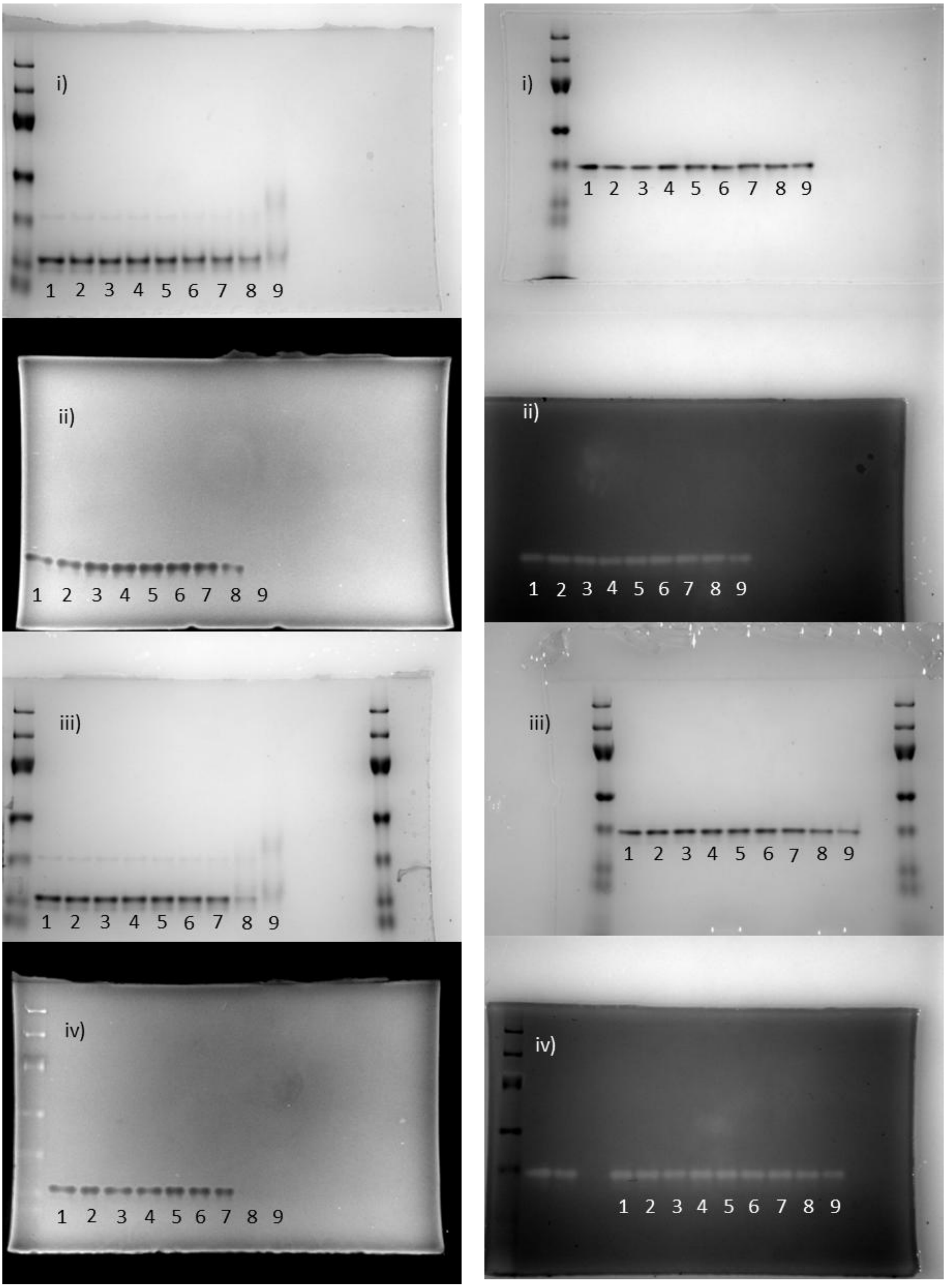

Supplementary Figure S7: Uncropped images. Raw data showing the unedited and uncropped gels from unfolding experiments shown in Fig. 2. Samples loaded were:

Mn-loaded MnSOD unfolding in urea

i) Coomassie stained native gel (280 ng of protein/well):

1) Mn-loaded MnSOD in 0.1 M urea; 2) Mn-loaded MnSOD in 0.5 M urea; 3) Mn-loaded MnSOD in 1.0 M urea; 4) Mn-loaded MnSOD in 1.5 M urea; 5) Mn-loaded MnSOD in 2.0 M urea; 6) Mn-loaded MnSOD in 2.5 M urea; 7) Mn-loaded MnSOD in 4 M urea; 8) Mn-loaded MnSOD in 6 M urea; 9) Mn-loaded MnSOD in 8 M urea

ii) SOD activity gel (17.5 ng of protein/well):

1) Mn-loaded MnSOD in 0.1 M urea; 2) Mn-loaded MnSOD in 0.5 M urea; 3) Mn-loaded MnSOD in 1.0 M urea; 4) Mn-loaded MnSOD in 1.5 M urea; 5) Mn-loaded MnSOD in 2.0 M urea; 6) Mn-loaded MnSOD in 2.5 M urea; 7) Mn-loaded MnSOD in 4 M urea; 8) Mn-loaded MnSOD in 6 M urea; 9) Mn-loaded MnSOD in 8 M urea

iii) Coomassie stained native gel with 50 mM EDTA (280 ng of protein/well):

1) Mn-loaded MnSOD in 0.1 M urea + 50 mM EDTA; 2) Mn-loaded MnSOD in 0.5 M urea + 50 mM EDTA; 3) Mn-loaded MnSOD in 1.0 M urea + 50 mM EDTA; 4) Mn-loaded MnSOD in 1.5 M urea + 50 mM EDTA; 5) Mn-loaded MnSOD in 2.0 M urea + 50 mM EDTA; 6) Mn-loaded MnSOD in 2.5 M urea + 50 mM EDTA; 7) Mn-loaded MnSOD in 4 M urea + 50 mM EDTA; 8) Mn-loaded MnSOD in 6 M urea + 50 mM EDTA; 9) Mn-loaded MnSOD in 8 M urea + 50 mM EDTA

iv) SOD activity gel with 50 mM EDTA (17.5 ng of protein/well):

1) Mn-loaded MnSOD in 0.1 M urea + 50 mM EDTA; 2) Mn-loaded MnSOD in 0.5 M urea + 50 mM EDTA; 3) Mn-loaded MnSOD in 1.0 M urea + 50 mM EDTA; 4) Mn-loaded MnSOD in 1.5 M urea + 50 mM EDTA; 5) Mn-loaded MnSOD in 2.0 M urea + 50 mM EDTA; 6) Mn-loaded MnSOD in 2.5 M urea + 50 mM EDTA; 7) Mn-loaded MnSOD in 4 M urea + 50 mM EDTA; 8) Mn-loaded MnSOD in 6 M urea + 50 mM EDTA; 9) Mn-loaded MnSOD in 8 M urea + 50 mM EDTA

Mn-loaded camSOD unfolding in urea

i) Coomassie stained native gel (280 ng of protein/well):

1) Mn-loaded camSOD in 0.1 M urea; 2) Mn-loaded camSOD in 0.5 M urea; 3) Mn-loaded camSOD in 1.0 M urea; 4) Mn-loaded camSOD in 1.5 M urea; 5) Mn-loaded camSOD in 2.0 M urea; 6) Mn-loaded camSOD in 2.5 M urea; 7) Mn-loaded camSOD in 4 M urea; 8) Mn-loaded camSOD in 6 M urea; 9) Mn-loaded camSOD in 8 M urea

ii) SOD activity gel (33 ng of protein/well):

1) Mn-loaded camSOD in 0.1 M urea; 2) Mn-loaded camSOD in 0.5 M urea; 3) Mn-loaded camSOD in 1.0 M urea; 4) Mn-loaded camSOD in 1.5 M urea; 5) Mn-loaded camSOD in 2.0 M urea; 6) Mn-loaded camSOD in 2.5 M urea; 7) Mn-loaded camSOD in 4 M urea; 8) Mn-loaded camSOD in 6 M urea; 9) Mn-loaded camSOD in 8 M urea

iii) Coomassie stained native gel with 50 mM EDTA (280 ng of protein/well):

1) Mn-loaded camSOD in 0.1 M urea + 50 mM EDTA; 2) Mn-loaded camSOD in 0.5 M urea + 50 mM EDTA; 3) Mn-loaded camSOD in 1.0 M urea + 50 mM EDTA; 4) Mn-loaded camSOD in 1.5 M urea + 50 mM EDTA; 5) Mn-loaded camSOD in 2.0 M urea + 50 mM EDTA; 6) Mn-loaded camSOD in 2.5 M urea + 50 mM EDTA; 7) Mn-loaded camSOD in 4 M urea + 50 mM EDTA; 8) Mn-loaded camSOD in 6 M urea + 50 mM EDTA; 9) Mn-loaded camSOD in 8 M urea + 50 mM EDTA

iv) SOD activity gel with 50 mM EDTA (33 ng of protein/well):

1) Mn-loaded camSOD in 0.1 M urea + 50 mM EDTA; 2) Mn-loaded camSOD in 0.5 M urea + 50 mM EDTA; 3) Mn-loaded camSOD in 1.0 M urea + 50 mM EDTA; 4) Mn-loaded camSOD in 1.5 M urea + 50 mM EDTA; 5) Mn-loaded camSOD in 2.0 M urea + 50 mM EDTA; 6) Mn-loaded camSOD in 2.5 M urea + 50 mM EDTA; 7) Mn-loaded camSOD in 4 M urea + 50 mM EDTA; 8) Mn-loaded camSOD in 6 M urea + 50 mM EDTA; 9) Mn-loaded camSOD in 8 M urea + 50 mM EDTA

Raw data uncropped gels –
Mn-loaded MnSOD unfolding in Gdn-HCl

Raw data uncropped gels –
Mn-loaded camSOD unfolding in Gdn-HCl

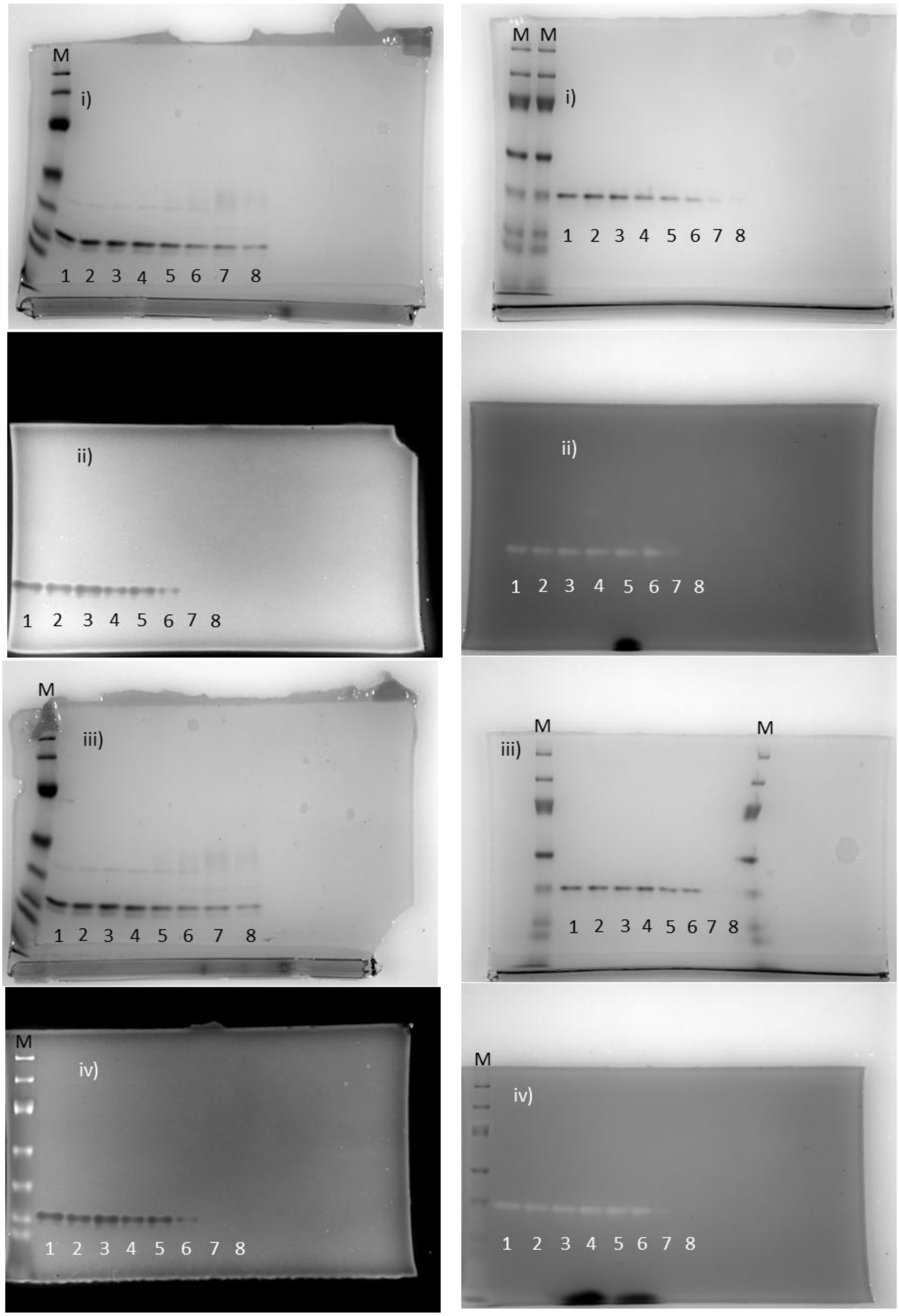

Supplementary Figure S8: Uncropped images. Raw data showing the unedited and uncropped gels from unfolding experiments shown in Fig. 3. Samples loaded were:

Mn-loaded MnSOD unfolding in GdnHCl

i) Coomassie stained native gel (280 ng of protein/well):

1) Mn-loaded MnSOD in 0.1 M GdnHCl; 2) Mn-loaded MnSOD in 0.5 M GdnHCl; 3) Mn-loaded MnSOD in 1.0 M GdnHCl; 4) Mn-loaded MnSOD in 1.5 M GdnHCl; 5) Mn-loaded MnSOD in 2.0 M GdnHCl; 6) Mn-loaded MnSOD in 2.5 M GdnHCl; 7) Mn-loaded MnSOD in 4 M GdnHCl; 8) Mn-loaded MnSOD in 6 M GdnHCl
ii) SOD activity gel (17.5 ng of protein/well):

1) Mn-loaded MnSOD in 0.1 M GdnHCl; 2) Mn-loaded MnSOD in 0.5 M GdnHCl; 3) Mn-loaded MnSOD in 1.0 M GdnHCl; 4) Mn-loaded MnSOD in 1.5 M GdnHCl; 5) Mn-loaded MnSOD in 2.0 M GdnHCl; 6) Mn-loaded MnSOD in 2.5 M GdnHCl; 7) Mn-loaded MnSOD in 4 M GdnHCl; 8) Mn-loaded MnSOD in 6 M GdnHCl
iii) Coomassie stained native gel with 50 mM EDTA (280 ng of protein/well):

1) Mn-loaded MnSOD in 0.1 M GdnHCl + 50 mM EDTA; 2) Mn-loaded MnSOD in 0.5 M GdnHCl + 50 mM EDTA; 3) Mn-loaded MnSOD in 1.0 M GdnHCl + 50 mM EDTA; 4) Mn-loaded MnSOD in 1.5 M GdnHCl + 50 mM EDTA; 5) Mn-loaded MnSOD in 2.0 M GdnHCl + 50 mM EDTA; 6) Mn-loaded MnSOD in 2.5 M GdnHCl + 50 mM EDTA; 7) Mn-loaded MnSOD in 4 M GdnHCl + 50 mM EDTA; 8) Mn-loaded MnSOD in 6 M GdnHCl + 50 mM EDTA
iv) SOD activity gel with 50 mM EDTA (17.5 ng of protein/well):

1) Mn-loaded MnSOD in 0.1 M GdnHCl + 50 mM EDTA; 2) Mn-loaded MnSOD in 0.5 M GdnHCl + 50 mM EDTA; 3) Mn-loaded MnSOD in 1.0 M GdnHCl + 50 mM EDTA; 4) Mn-loaded MnSOD in 1.5 M GdnHCl + 50 mM EDTA; 5) Mn-loaded MnSOD in 2.0 M GdnHCl + 50 mM EDTA; 6) Mn-loaded MnSOD in 2.5 M GdnHCl + 50 mM EDTA; 7) Mn-loaded MnSOD in 4 M GdnHCl + 50 mM EDTA; 8) Mn-loaded MnSOD in 6 M GdnHCl + 50 mM EDTA

Mn-loaded camSOD unfolding in GdnHCl

i) Coomassie stained native gel (280 ng of protein/well):

1) Mn-loaded camSOD in 0.1 M GdnHCl; 2) Mn-loaded camSOD in 0.5 M GdnHCl; 3) Mn-loaded camSOD in 1.0 M GdnHCl; 4) Mn-loaded camSOD in 1.5 M GdnHCl; 5) Mn-loaded camSOD in 2.0 M GdnHCl; 6) Mn-loaded camSOD in 2.5 M GdnHCl; 7) Mn-loaded camSOD in 4 M GdnHCl; 8) Mn-loaded camSOD in 6 M GdnHCl
ii) SOD activity gel (33 ng of protein/well):

1) Mn-loaded camSOD in 0.1 M GdnHCl; 2) Mn-loaded camSOD in 0.5 M GdnHCl; 3) Mn-loaded camSOD in 1.0 M GdnHCl; 4) Mn-loaded camSOD in 1.5 M GdnHCl; 5) Mn-loaded camSOD in 2.0 M GdnHCl; 6) Mn-loaded camSOD in 2.5 M GdnHCl; 7) Mn-loaded camSOD in 4 M GdnHCl; 8) Mn-loaded camSOD in 6 M GdnHCl
iii) Coomassie stained native gel with 50 mM EDTA (280 ng of protein/well):

1) Mn-loaded camSOD in 0.1 M GdnHCl + 50 mM EDTA; 2) Mn-loaded camSOD in 0.5 M GdnHCl + 50 mM EDTA; 3) Mn-loaded camSOD in 1.0 M GdnHCl + 50 mM EDTA; 4) Mn-loaded camSOD in 1.5 M GdnHCl + 50 mM EDTA; 5) Mn-loaded camSOD in 2.0 M GdnHCl + 50 mM EDTA; 6) Mn-loaded camSOD in 2.5 M GdnHCl + 50 mM EDTA; 7) Mn-loaded camSOD in 4 M GdnHCl + 50 mM EDTA; 8) Mn-loaded camSOD in 6 M GdnHCl + 50 mM EDTA
iv) SOD activity gel with 50 mM EDTA (33 ng of protein/well):

1) Mn-loaded camSOD in 0.1 M GdnHCl + 50 mM EDTA; 2) Mn-loaded camSOD in 0.5 M GdnHCl + 50 mM EDTA; 3) Mn-loaded camSOD in 1.0 M GdnHCl + 50 mM EDTA; 4) Mn-loaded camSOD in 1.5 M GdnHCl + 50 mM EDTA; 5) Mn-loaded camSOD in 2.0 M GdnHCl + 50 mM EDTA; 6) Mn-loaded camSOD in 2.5 M GdnHCl + 50 mM EDTA; 7) Mn-loaded camSOD in 4 M GdnHCl + 50 mM EDTA; 8) Mn-loaded camSOD in 6 M GdnHCl + 50 mM EDTA
M stands for molecular weight markers (Prestained Protein Ladder from Thermo Scientific)

Raw data uncropped gels –
Fe-loaded MnSOD unfolding in urea

Raw data uncropped gels –
Fe-loaded camSOD unfolding in urea

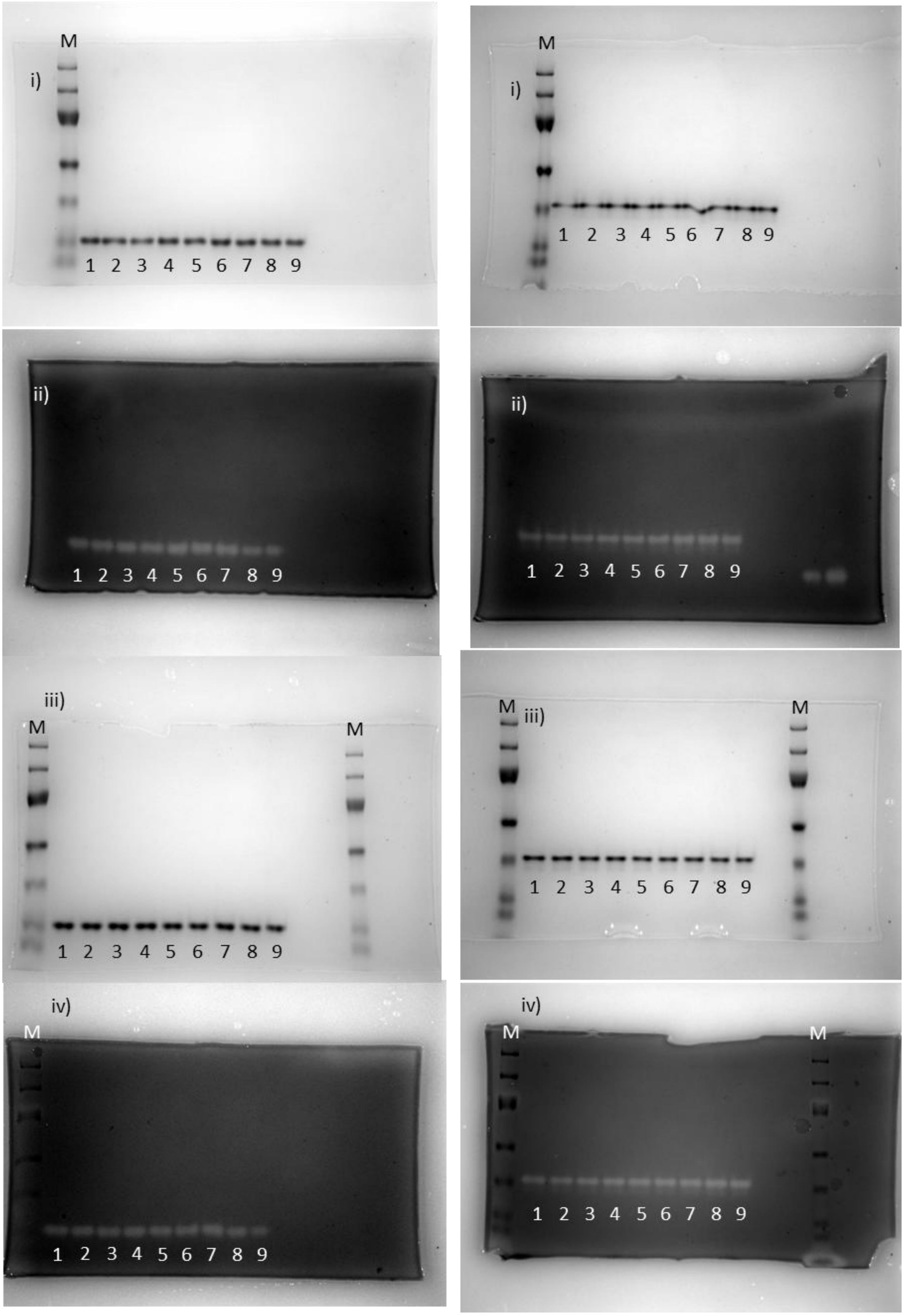

Supplementary Figure S9: Uncropped images. Raw data showing the unedited and uncropped gels from unfolding experiments shown in Supp. Fig. S4. Samples loaded were:

Fe-loaded MnSOD unfolding in urea

i) Coomassie stained native gel (280 ng of protein/well):

1) Fe-loaded MnSOD in 0.1 M urea; 2) Fe-loaded MnSOD in 0.5 M urea; 3) Fe-loaded MnSOD in 1.0 M urea; 4) Fe-loaded MnSOD in 1.5 M urea; 5) Fe-loaded MnSOD in 2.0 M urea; 6) Fe-loaded MnSOD in 2.5 M urea; 7) Fe-loaded MnSOD in 4 M urea; 8) Fe-loaded MnSOD in 6 M urea; 9) Fe-loaded MnSOD in 8 M urea

ii) SOD activity gel (698.3 ng of protein/well):

1) Fe-loaded MnSOD in 0.1 M urea; 2) Fe-loaded MnSOD in 0.5 M urea; 3) Fe-loaded MnSOD in 1.0 M urea; 4) Fe-loaded MnSOD in 1.5 M urea; 5) Fe-loaded MnSOD in 2.0 M urea; 6) Fe-loaded MnSOD in 2.5 M urea; 7) Fe-loaded MnSOD in 4 M urea; 8) Fe-loaded MnSOD in 6 M urea; 9) Fe-loaded MnSOD in 8 M urea

iii) Coomassie stained native gel with 50 mM EDTA (280 ng of protein/well):

1) Fe-loaded MnSOD in 0.1 M urea + 50 mM EDTA; 2) Fe-loaded MnSOD in 0.5 M urea + 50 mM EDTA; 3) Fe-loaded MnSOD in 1.0 M urea + 50 mM EDTA; 4) Fe-loaded MnSOD in 1.5 M urea + 50 mM EDTA; 5) Fe-loaded MnSOD in 2.0 M urea + 50 mM EDTA; 6) Fe-loaded MnSOD in 2.5 M urea + 50 mM EDTA; 7) Fe-loaded MnSOD in 4 M urea + 50 mM EDTA; 8) Fe-loaded MnSOD in 6 M urea + 50 mM EDTA; 9) Fe-loaded MnSOD in 8 M urea + 50 mM EDTA

iv) SOD activity gel with 50 mM EDTA (698.3 ng of protein/well):

1) Fe-loaded MnSOD in 0.1 M urea + 50 mM EDTA; 2) Fe-loaded MnSOD in 0.5 M urea + 50 mM EDTA; 3) Fe-loaded MnSOD in 1.0 M urea + 50 mM EDTA; 4) Fe-loaded MnSOD in 1.5 M urea + 50 mM EDTA; 5) Fe-loaded MnSOD in 2.0 M urea + 50 mM EDTA; 6) Fe-loaded MnSOD in 2.5 M urea + 50 mM EDTA; 7) Fe-loaded MnSOD in 4 M urea + 50 mM EDTA; 8) Fe-loaded MnSOD in 6 M urea + 50 mM EDTA; 9) Fe-loaded MnSOD in 8 M urea + 50 mM EDTA

Fe-loaded camSOD unfolding in urea

i) Coomassie stained native gel (280 ng of protein/well):

1) Fe-loaded camSOD in 0.1 M urea; 2) Fe-loaded camSOD in 0.5 M urea; 3) Fe-loaded camSOD in 1.0 M urea; 4) Fe-loaded camSOD in 1.5 M urea; 5) Fe-loaded camSOD in 2.0 M urea; 6) Fe-loaded camSOD in 2.5 M urea; 7) Fe-loaded camSOD in 4 M urea; 8) Fe-loaded camSOD in 6 M urea; 9) Fe-loaded camSOD in 8 M urea

ii) SOD activity gel (41.4 ng of protein/well):

1) Fe-loaded camSOD in 0.1 M urea; 2) Fe-loaded camSOD in 0.5 M urea; 3) Fe-loaded camSOD in 1.0 M urea; 4) Fe-loaded camSOD in 1.5 M urea; 5) Fe-loaded camSOD in 2.0 M urea; 6) Fe-loaded camSOD in 2.5 M urea; 7) Fe-loaded camSOD in 4 M urea; 8) Fe-loaded camSOD in 6 M urea; 9) Fe-loaded camSOD in 8 M urea

iii) Coomassie stained native gel with 50 mM EDTA (280 ng of protein/well):

1) Mn-loaded camSOD in 0.1 M urea + 50 mM EDTA; 2) Mn-loaded camSOD in 0.5 M urea + 50 mM EDTA; 3) Mn-loaded camSOD in 1.0 M urea + 50 mM EDTA; 4) Mn-loaded camSOD in 1.5 M urea + 50 mM EDTA; 5) Mn-loaded camSOD in 2.0 M urea + 50 mM EDTA; 6) Mn-loaded camSOD in 2.5 M urea + 50 mM EDTA; 7) Mn-loaded camSOD in 4 M urea + 50 mM EDTA; 8) Mn-loaded camSOD in 6 M urea + 50 mM EDTA; 9) Mn-loaded camSOD in 8 M urea + 50 mM EDTA

iv) SOD activity gel with 50 mM EDTA (41.4 ng of protein/well):

1) Fe-loaded camSOD in 0.1 M urea + 50 mM EDTA; 2) Fe-loaded camSOD in 0.5 M urea + 50 mM EDTA; 3) Fe-loaded camSOD in 1.0 M urea + 50 mM EDTA; 4) Fe-loaded camSOD in 1.5 M urea + 50 mM EDTA; 5) Fe-loaded camSOD in 2.0 M urea + 50 mM EDTA; 6) Fe-loaded camSOD in 2.5 M urea + 50 mM EDTA; 7) Fe-loaded camSOD in 4 M urea + 50 mM EDTA; 8) Fe-loaded camSOD in 6 M urea + 50 mM EDTA; 9) Fe-loaded camSOD in 8 M urea + 50 mM EDTA

Raw data uncropped gels –
Fe-loaded MnSOD unfolding in gdn-HCl

Raw data uncropped gels –
Fe-loaded camSOD unfolding in gdn-HCl

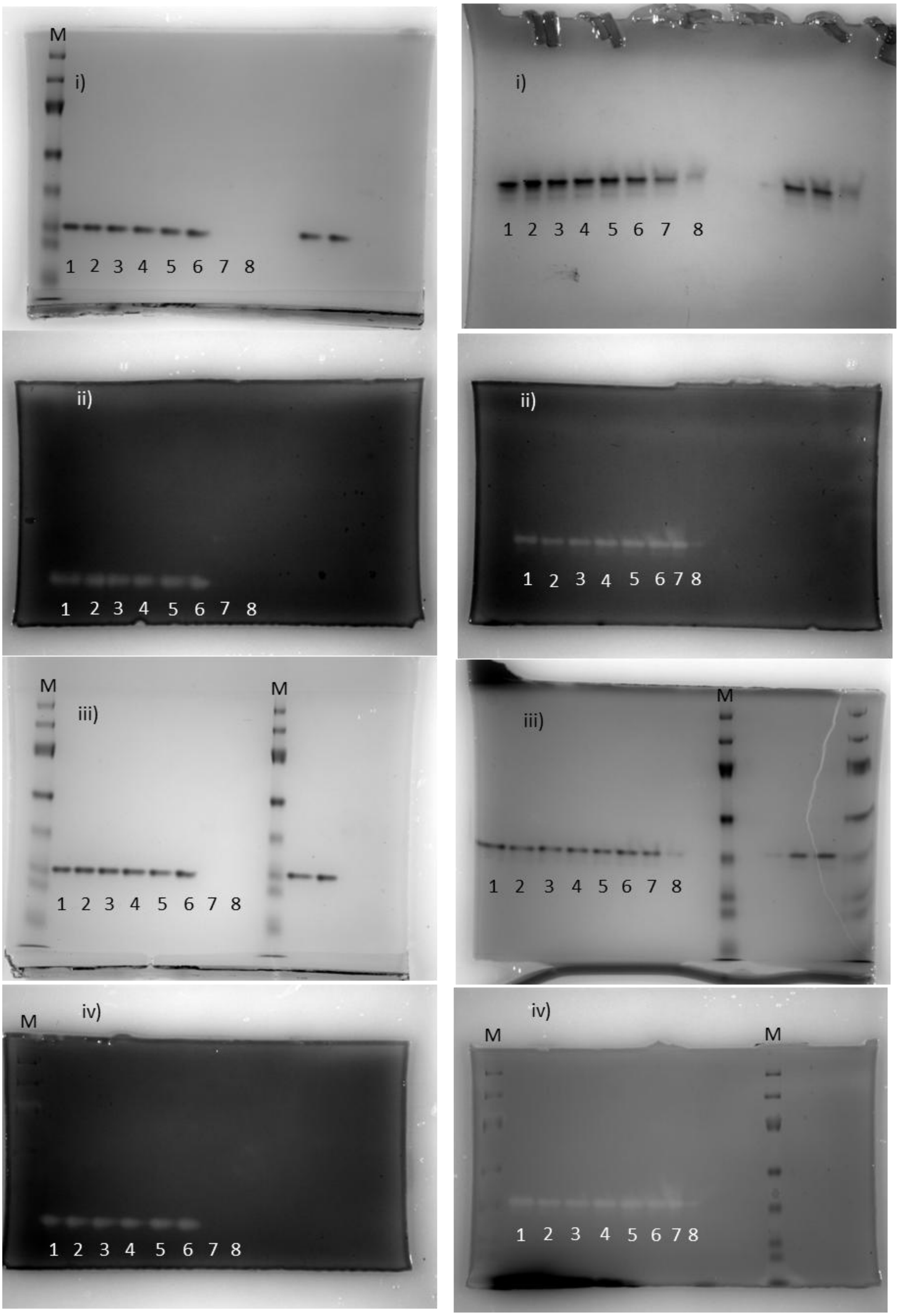

Supplementary Figure S10: Uncropped images. Raw data showing the unedited and uncropped gels from unfolding experiments shown in Supp. Fig. S5. Samples loaded were:

Fe-loaded MnSOD unfolding in GdnHCl

i) Coomassie stained native gel (280 ng of protein/well):

1) Fe-loaded MnSOD in 0.1 M GdnHCl; 2) Fe-loaded MnSOD in 0.5 M GdnHCl; 3) Fe-loaded MnSOD in 1.0 M GdnHCl; 4) Fe-loaded MnSOD in 1.5 M GdnHCl; 5) Fe-loaded MnSOD in 2.0 M GdnHCl; 6) Fe-loaded MnSOD in 2.5 M GdnHCl; 7) Fe-loaded MnSOD in 4 M GdnHCl; 8) Fe-loaded MnSOD in 6 M GdnHCl
ii) SOD activity gel (698.3 ng of protein/well):

1) Fe-loaded MnSOD in 0.1 M GdnHCl; 2) Fe-loaded MnSOD in 0.5 M gdn-HCl; 3) Fe-loaded MnSOD in 1.0 M GdnHCl; 4) Fe-loaded MnSOD in 1.5 M GdnHCl; 5) Fe-loaded MnSOD in 2.0 M GdnHCl; 6) Fe-loaded MnSOD in 2.5 M GdnHCl; 7) Fe-loaded MnSOD in 4 M GdnHCl; 8) Fe-loaded MnSOD in 6 M GdnHCl
iii) Coomassie stained native gel with 50 mM EDTA (280 ng of protein/well):

1) Fe-loaded MnSOD in 0.1 M GdnHCl + 50 mM EDTA; 2) Fe-loaded MnSOD in 0.5 M GdnHCl + 50 mM EDTA; 3) Fe-loaded MnSOD in 1.0 M GdnHCl + 50 mM EDTA; 4) Fe-loaded MnSOD in 1.5 M GdnHCl + 50 mM EDTA; 5) Fe-loaded MnSOD in 2.0 M GdnHCl + 50 mM EDTA; 6) Fe-loaded MnSOD in 2.5 M GdnHCl + 50 mM EDTA; 7) Fe-loaded MnSOD in 4.0 M GdnHCl + 50 mM EDTA; 8) Fe-loaded MnSOD in 6.0 M GdnHCl + 50 mM EDTA
iv) SOD activity gel with 50 mM EDTA (698.3 ng of protein/well):

1) Fe-loaded MnSOD in 0.1 M GdnHCl + 50 mM EDTA; 2) Fe-loaded MnSOD in 0.5 M GdnHCl + 50 mM EDTA; 3) Fe-loaded MnSOD in 1.0 M GdnHCl + 50 mM EDTA; 4) Fe-loaded MnSOD in 1.5 M GdnHCl + 50 mM EDTA; 5) Fe-loaded MnSOD in 2.0 M GdnHCl + 50 mM EDTA; 6) Fe-loaded MnSOD in 2.5 M GdnHCl + 50 mM EDTA; 7) Fe-loaded MnSOD in 4.0 M GdnHCl + 50 mM EDTA; 8) Fe-loaded MnSOD in 6.0 M GdnHCl + 50 mM EDTA

Fe-loaded camSOD unfolding in Gdn-HCl

i) Coomassie stained native gel (280 ng of protein/well):

1) Fe-loaded camSOD in 0.1 M GdnHCl; 2) Fe-loaded camSOD in 0.5 M GdnHCl; 3) Fe-loaded camSOD in 1.0 M GdnHCl; 4) Fe-loaded camSOD in 1.5 M GdnHCl; 5) Fe-loaded camSOD in 2.0 M GdnHCl; 6) Fe-loaded camSOD in 2.5 M GdnHCl; 7) Fe-loaded camSOD in 4 M GdnHCl; 8) Fe-loaded camSOD in 6 M GdnHCl
ii) SOD activity gel (41.4 ng of protein/well):

1) Fe-loaded camSOD in 0.1 M GdnHCl; 2) Fe-loaded camSOD in 0.5 M GdnHCl; 3) Fe-loaded camSOD in 1.0 M GdnHCl; 4) Fe-loaded camSOD in 1.5 M GdnHCl; 5) Fe-loaded camSOD in 2.0 M GdnHCl; 6) Fe-loaded camSOD in 2.5 M GdnHCl; 7) Fe-loaded camSOD in 4 M GdnHCl; 8) Fe-loaded camSOD in 6 M GdnHCl
iii) Coomassie stained native gel with 50 mM EDTA (280 ng of protein/well):

1) Fe-loaded camSOD in 0.1 M GdnHCl + 50 mM EDTA; 2) Fe-loaded camSOD in 0.5 M GdnHCl + 50 mM EDTA; 3) Mn- Fe-loaded camSOD SodM in 1.0 M GdnHCl + 50 mM EDTA; 4) Fe-loaded camSOD in 1.5 M GdnHCl + 50 mM EDTA; 5) Fe-loaded camSOD in 2.0 M GdnHCl + 50 mM EDTA; 6) Fe-loaded camSOD in 2.5 M GdnHCl + 50 mM EDTA; 7) Fe-loaded camSOD in 4 M GdnHCl + 50 mM EDTA; 8) Fe-loaded camSOD in 6 M GdnHCl + 50 mM EDTA
iv) SOD activity gel with 50 mM EDTA (41.4 ng of protein/well):

1) Fe-loaded camSOD in 0.1 M GdnHCl + 50 mM EDTA; 2) Fe-loaded camSOD in 0.5 M GdnHCl + 50 mM EDTA; 3) Fe-loaded camSOD in 1.0 M GdnHCl + 50 mM EDTA; 4) M Fe-loaded camSOD in 1.5 M GdnHCl + 50 mM EDTA; 5) Fe-loaded camSOD in 2.0 M GdnHCl + 50 mM EDTA; 6) Fe-loaded camSOD in 2.5 M GdnHCl + 50 mM EDTA; 7 Fe-loaded camSOD in 4 M GdnHCl + 50 mM EDTA; 8) Fe-loaded camSOD in 6 M GdnHCl + 50 mM EDTA

M stands for Molecular weight markers (Prestained Protein Ladder from Thermo Scientific)

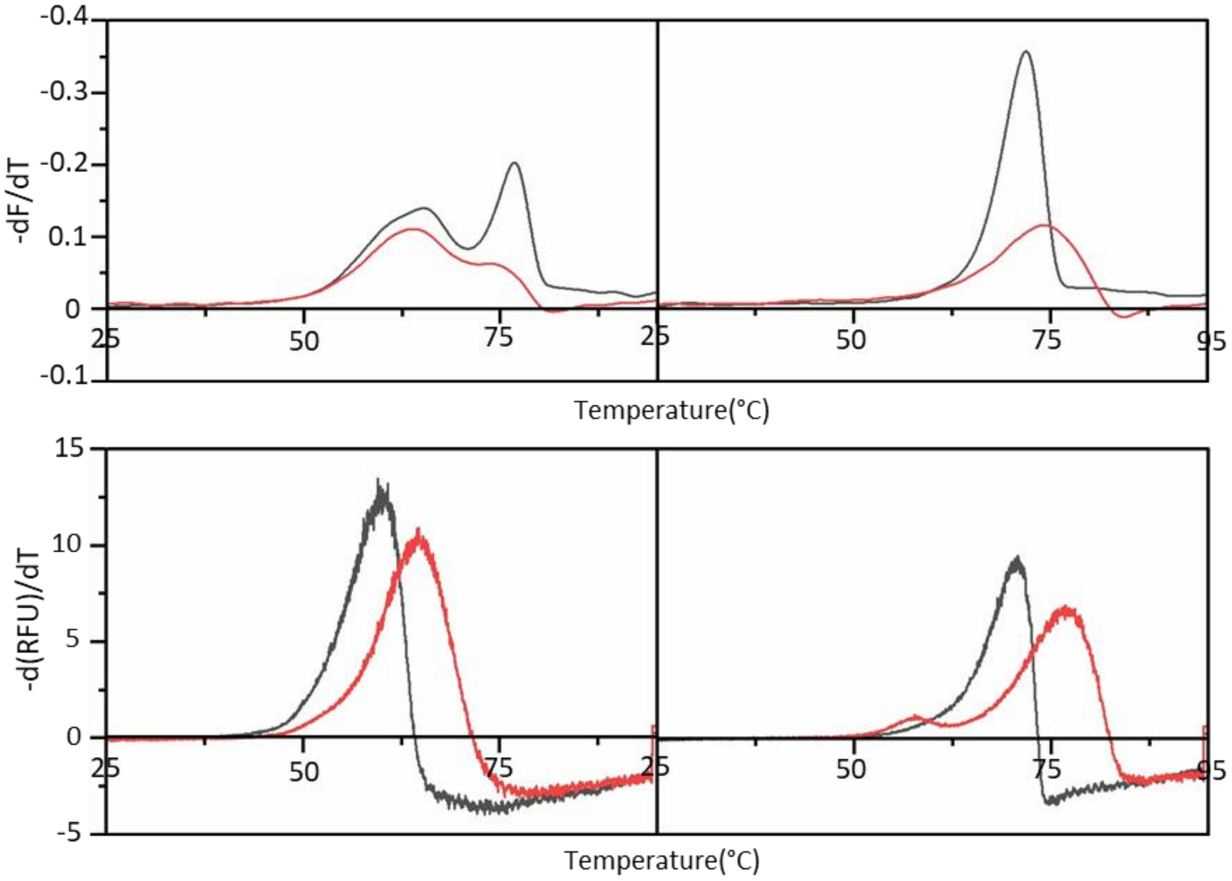

Supplementary Figure S11: First derivative of fluorescence thermal melting curves. First derivative curves of (upper panels) nano DSF data and (lower panels) Sypro Orange thermal assay data, illustrating the temperature-dependent structural transitions of (left) manganese-loaded and (right) iron-loaded isoforms of MnSOD (black) and camSOD (red).

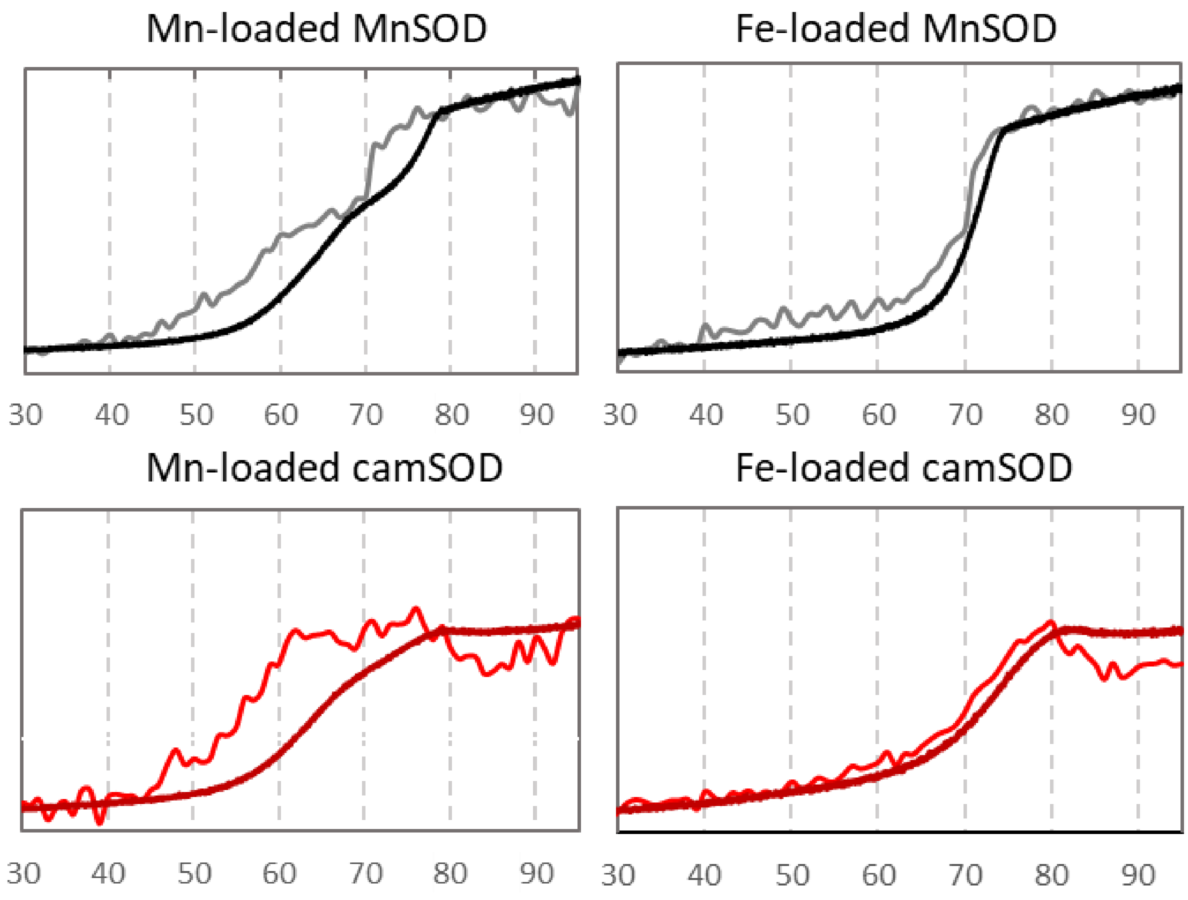

Supplementary Figure S12: Comparison of thermal melt curves obtained from CD spectroscopy and nano DSF. Overlaid data from CD spectroscopic analyses of protein thermal melting (thin lines), representing the deterioration of α-helical structural content of the proteins, and nano DSF analyses of protein thermal melting (thick lines), representing the decomposition of the hydrophobic core of the structure as represented by change in Trp fluorescence. The y-axes have been normalised to allow comparison.

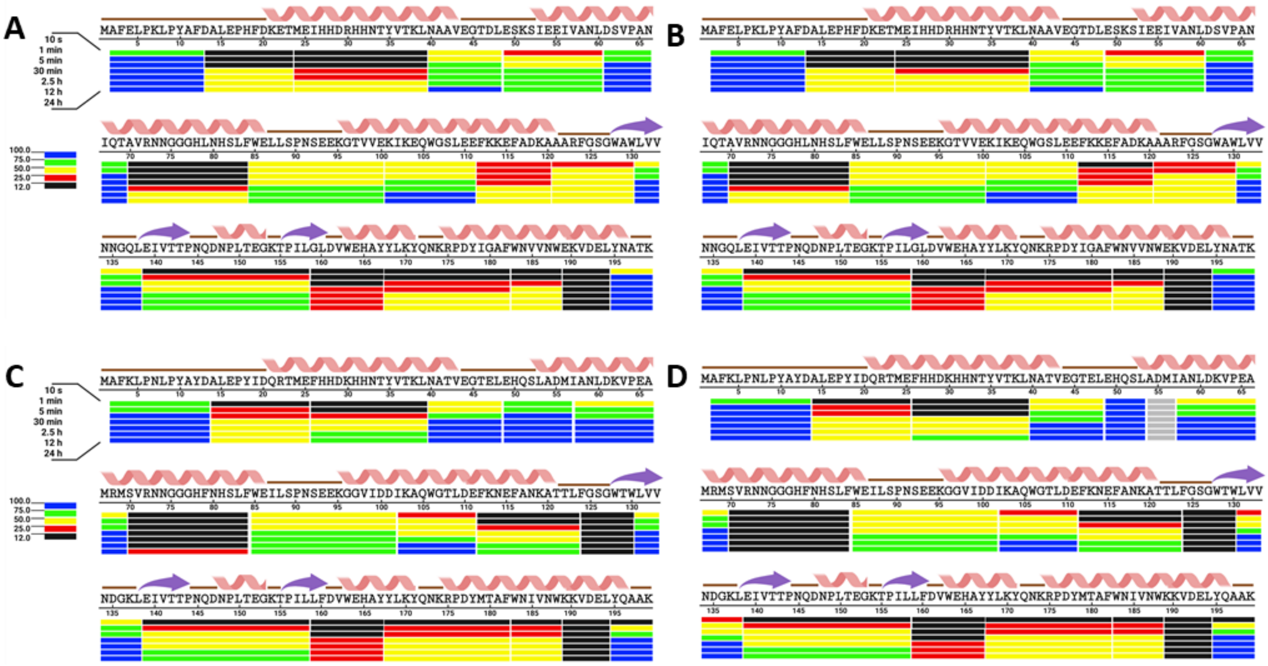

Supplementary Figure S13: Overview of rates of hydrogen-deuterium exchange across the length of the *S. aureus* SODs polypeptides. Overview of the pattern of rates of hydrogen-deuterium exchange observed within A) Mn-loaded MnSOD, B) Fe-loaded MnSOD, C) Mn-loaded camSOD, D) Fe-loaded camSOD. HDX-MS analysis of peptides was performed before (t=0 control) and after 10 s, 1 min, 5 min, 30 min, 2.5 h, 12 h, and 24 h exposure to deuterated solvent (see Supp. Fig. S11) identify differences in main chain amide exchange rates between the two isozymes. Representative peptides which enabled comparison between the MnSOD and camSOD isozymes were selected, and their rates of exchange were depicted in BioRender. The peptides are selected to provide maximum coverage of the protein while retaining the shortest peptide possible in the region. Color scheme shows the fraction of exchanged amide protons after a given incubation period, >12%: black; 12 – 25%: red; 25 -50%: yellow; 50 – 75%: green; 100%: blue.

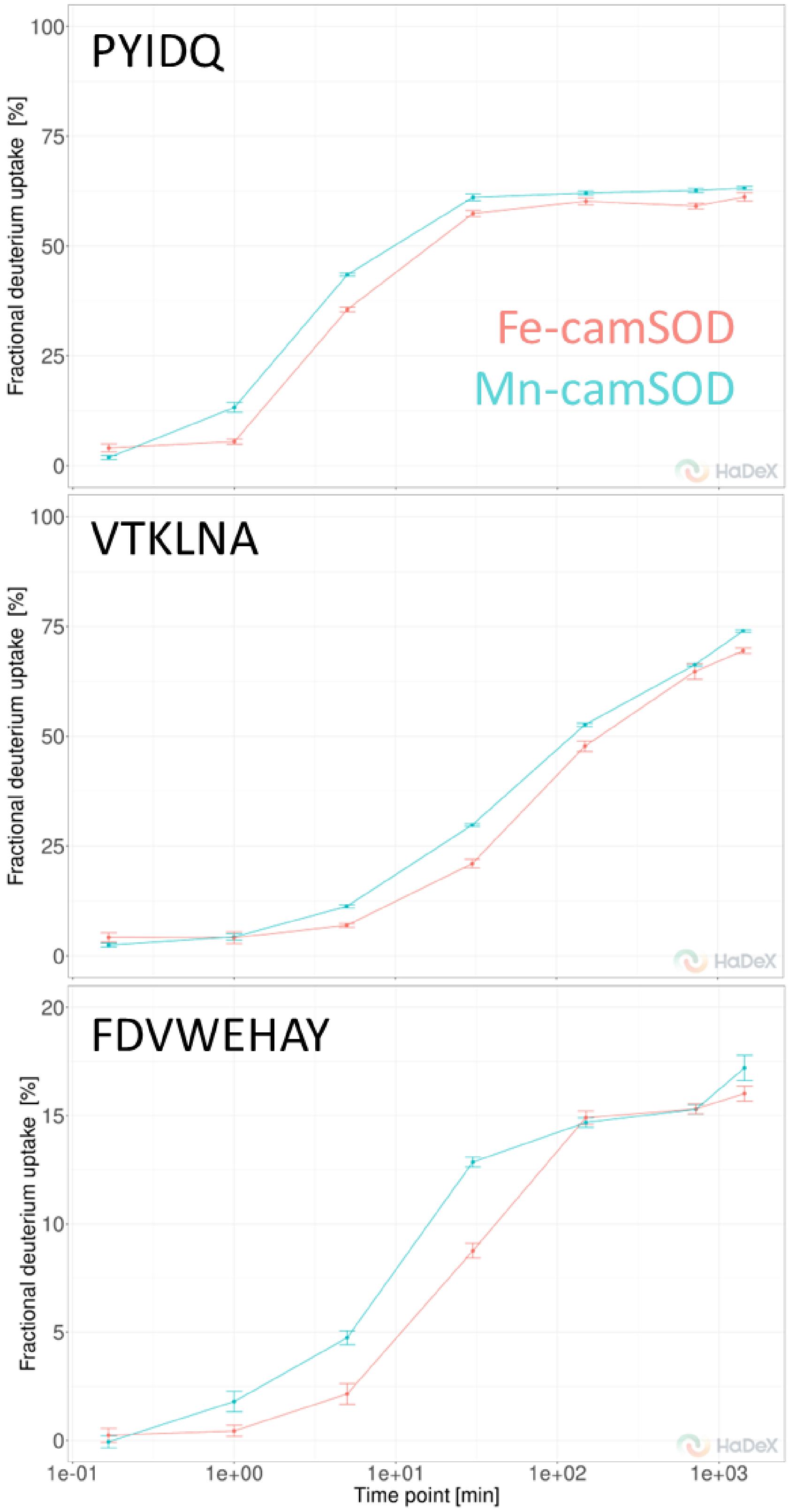

Supplementary Figure S14: Rates of amide hydrogen-deuterium exchange in specific peptides of *S. aureus* SodM. Deuterium uptake traces for three selected peptides from SodM, with data illustrating the extent of deuterium uptake in the iron-loaded (pink) and manganese-loaded (teal) form. These peptides were selected to demonstrate that some peptides did show detectable, quantitative differences in their rate of deuterium uptake between the different metal-loaded isoforms of the SodFM proteins. However, these differences were not as large as the differences observed between isozymes. The HDX-MS dataset has been deposited in the PRIDE repository under accession number PXD057066 and is publicly accessible.

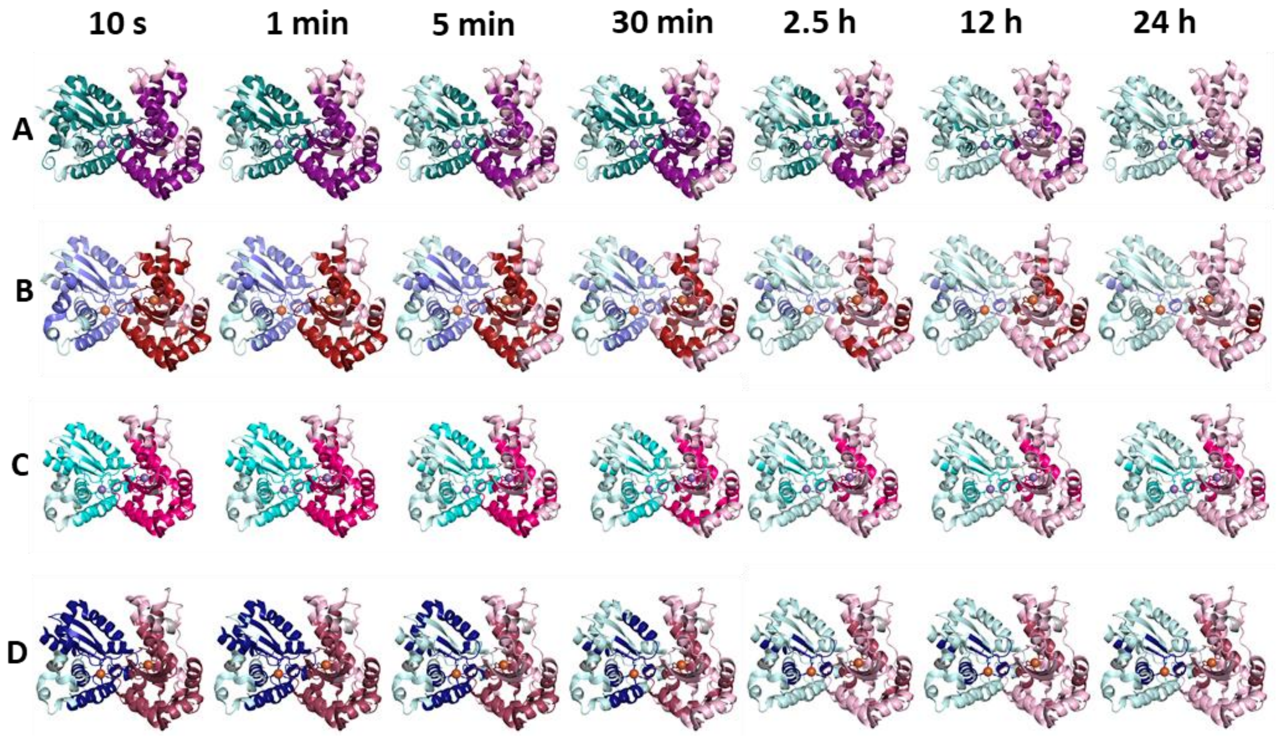

Supplementary Figure S15: Overview of rates of hydrogen-deuterium exchange across the length of the *S. aureus* SODs polypeptides. Structural illustration, demonstrating regions of the SodFM structures of A: Mn-loaded MnSOD, B: Fe-loaded MnSOD, C: Mn-loaded camSOD, D: Fe-loaded camSOD that exhibited low rates of deuterium exchange of their main-chain amide hydrogen atoms in HDX-MS analyses. Peptide regions that exhibited low overall deuteration (30% cut off) at each time was shown (dark coloured ribbons) on the overall structural models (light coloured ribbons) to illustrate which regions of each isozyme's structure was resistant to main-chain amide proton exchange. The values to represent in the structure are obtained for each residue based on the uptakes from overlapping peptides using the weighted approach in DynamX 3.0).

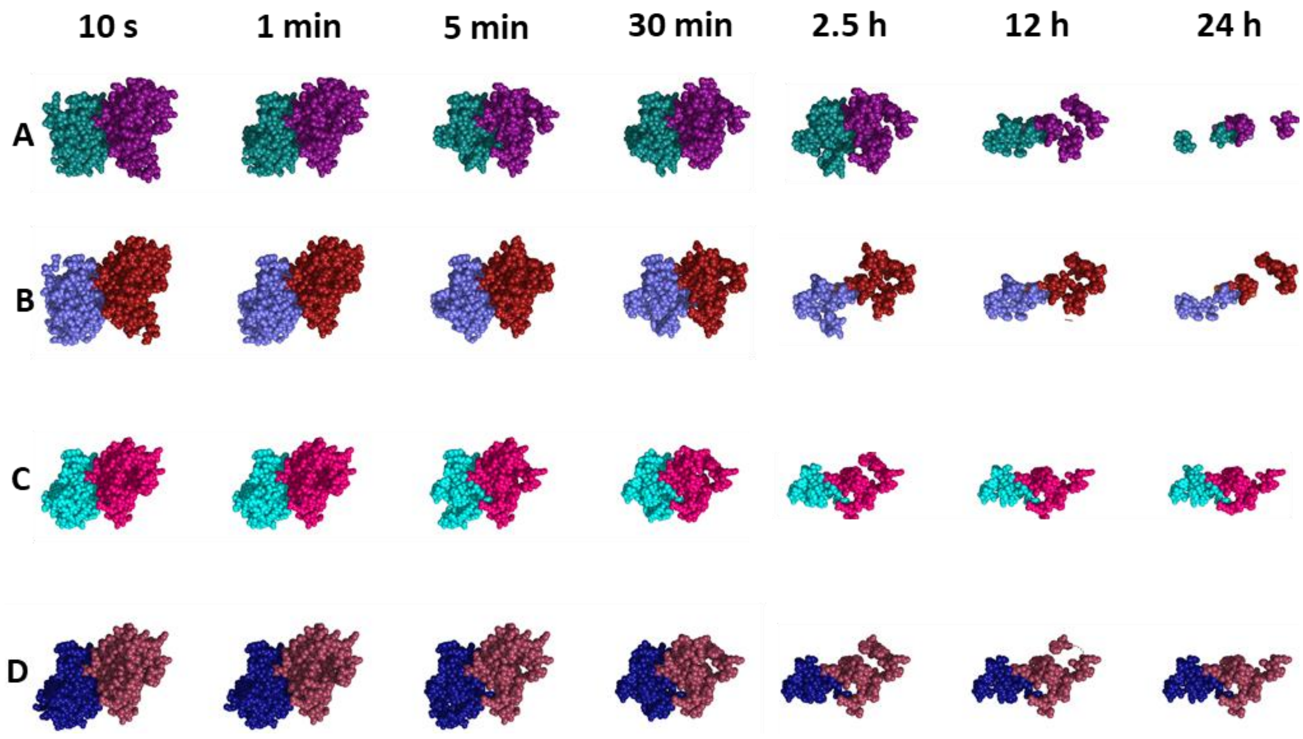

Supplementary Figure S16: Overview of rates of hydrogen-deuterium exchange across the length of the *S. aureus* SODs polypeptides. Alternative structural illustration, demonstrating regions of the SodFM structures of A: Mn-loaded MnSOD, B: Fe-loaded MnSOD, C: Mn-loaded camSOD, D: Fe-loaded camSOD that exhibited low rates of deuterium exchange of their main-chain amide hydrogen atoms in HDX-MS analyses. Peptide regions that exhibited low overall deuteration (30% cut off) at each time was shown in space-filling representation to illustrate the volume of the regions of each isozyme's structure that was resistant to main-chain amide proton exchange. The values to represent in the structure are obtained for each residue based on the uptakes from overlapping peptides using the weighted approach in DynamX 3.0).

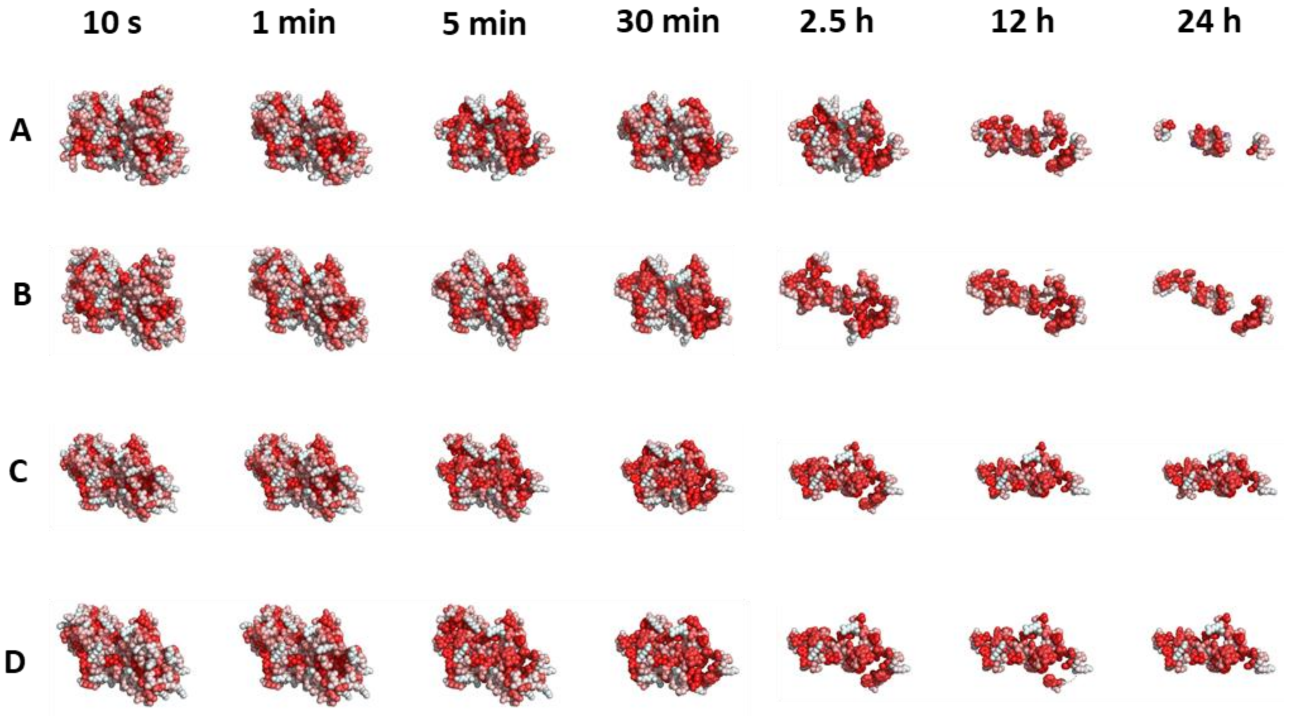

Supplementary Figure S17: Overview of rates of hydrogen-deuterium exchange across the length of the *S. aureus* SODs polypeptides. Alternative structural illustration, demonstrating regions of the SodFM structures of A: Mn-loaded MnSOD, B: Fe-loaded MnSOD, C: Mn-loaded camSOD, D: Fe-loaded camSOD that exhibited low rates of deuterium exchange of their main-chain amide hydrogen atoms in HDX-MS analyses. Peptide regions that exhibited low overall deuteration (30% cut off) at each time was shown in space-filling representation, coloured according to hydrophobicity in Pymol, as an alternative representation of the volume of the regions of each isozyme's structure that was resistant to main-chain amide proton exchange. The values to represent in the structure are obtained for each residue based on the uptakes from overlapping peptides using the weighted approach in DynamX 3.0).
